# Newer insights into the mechanism of action of *Psidium guajava *L. leaves in infectious diarrhoea

**DOI:** 10.1186/1472-6882-10-33

**Published:** 2010-06-28

**Authors:** Tannaz Birdi, Poonam Daswani, S Brijesh, Pundarikakshudu Tetali, Arvind Natu, Noshir Antia

**Affiliations:** 1 The Foundation for Medical Research, 84A, RG Thadani Marg, Worli, Mumbai 400018, Maharashtra, India; 2 Naoroji Godrej Centre for Plant Research, Lawkin Ltd. Campus, Shindewadi, Shirwal, Satara 412801, Maharashtra, India; 3 Indian Institute of Science Education and Research, Central Tower, Sai Trinity, Garware Circle, Sutarwadi, Pashan, Pune 411021, Maharashtra, India; 4 The Foundation for Research in Community Health, 3-4, Trimiti-B Apartments, 85, Anand Park, Pune 411 007, Maharashtra, India

## Abstract

**Background:**

*Psidium guajava *L., Myrtaceae, is used widely in traditional medicine for the treatment of diarrhoea, dysentery, gastroenteritis, stomachaches, and indigestion. However, the effect of the leaf extract of *P. guajava *on the pathogenesis of infectious diarrhoea has not been studied. The present study evaluates the effect of a hot aqueous extract (decoction) of dried leaves of *P. guajava *on parameters associated with pathogenicity of infectious diarrhoea. The aim was to understand its possible mechanism(s) of action in controlling infectious diarrhoea and compare it with quercetin, one of the most reported active constituents of *P. guajava *with antidiarrhoeal activity.

**Methods:**

The crude decoction and quercetin were studied for their antibacterial activity and effect on virulence features of common diarrhoeal pathogens viz. colonization of epithelial cells and production and action of enterotoxins. Colonization as measured by adherence of enteropathogenic *Escherichia coli *(EPEC) and invasion of enteroinvasive *E. coli *(EIEC) and *Shigella flexneri *was assessed using HEp-2 cell line. The production of *E. coli *heat labile toxin (LT) and cholera toxin (CT) and their binding to ganglioside monosialic acid (GM1) were studied by GM1-ELISA whereas the production and action of *E. coli *heat stable toxin (ST) was assessed by suckling mouse assay.

**Results:**

The decoction of *P. guajava *showed antibacterial activity towards *S. flexneri *and *Vibrio cholerae*. It decreased production of both LT and CT and their binding to GM1. However, it had no effect on production and action of ST. The decoction also inhibited the adherence of EPEC and invasion by both EIEC and *S. flexneri *to HEp-2 cells. Quercetin, on the other hand, had no antibacterial activity at the concentrations used nor did it affect any of the enterotoxins. Although it did not affect adherence of EPEC, it inhibited the invasion of both EIEC and *S. flexneri *to HEp-2 cells.

**Conclusion:**

Collectively, the results indicate that the decoction of *P. guajava *leaves is an effective antidiarrhoeal agent and that the entire spectrum of its antidiarrhoeal activity is not due to quercetin alone.

## Background

Infectious diarrhoea accounts for nearly 3.2% of all deaths globally [1] and is the second largest cause of years of productive life lost due to premature mortality and disability [2]. It is a major health concern in developing countries and remains an important clinical problem even in developed countries despite improvements in public health and economic wealth [3]. It is estimated that during the next 20-30 years, diarrhoea along with other infectious diseases will remain a cause of global health concern [4].

Diarrhoea is an etiologically diverse condition unlike some other infectious diseases such as tuberculosis, HIV/AIDS and malaria since it is caused by a variety of enteric pathogens including bacteria, viruses and protozoa [5-7].

Oral rehydration therapy has been the key strategy for effective case management. However, it often fails in high stool output state. Moreover, symptomatic therapy with antimotility agents is contraindicated in infectious diarrhoea and there is an increasing threat of drug resistance to antibiotics [8]. Various attempts for developing vaccines against diarrhoea causing organisms have been made [6,9-11]. However, the responses to vaccines in developing countries have not been encouraging [12-15]. In the recent past there have been advances towards the treatment of infectious diarrhoea with supportive therapy such as the use of probiotics; but these are still under development [3]. Hence, medicinal plants may aid in developing cost effective alternative approaches for treatment of diarrhoea.

Medicinal plants have recently gained popularity as prospective antidiarrhoeal agents as can be judged by the number of studies that have been undertaken. An online search on PubMed shows that more than 200 studies on the antidiarrhoeal activity of medicinal plants have been published in the last decade. Gutierrez *et al*. [16] have reviewed more than 50 such studies conducted during the period 2000 to 2007. Whilst a few studies have reported antimicrobial activity, a majority of the studies have focused on the effect of the plants on intestinal motility in experimental models. Hence, though data is available on the effect of medicinal plants on physiological diarrhoea as studied in animal models there is a paucity of information on their mode of action on infectious diarrhoea.

*Psidium guajava *L., Myrtaceae, is used widely in traditional medicine throughout Latin America and the Caribbean for the treatment of diarrhoea, dysentery, gastroenteritis, stomachaches, and indigestion [17]. It is also used for diarrhoea and dysentery in countries such as China, Philippines, Senegal and USA, as an antiamoebic in Congo, antispasmodic in India and Ghana, antiseptic in China and as an antibiotic in USA. However, this plant is not very popular in India as an antidiarrhoeal agent. An ethnobotanical survey carried out by us in Parinche valley located 55 km south-east of Pune, Maharashtra, India revealed that *P. guajava *had very limited usage by the local community as a treatment for diarrhoea [18]. Only 2 out of 24 traditional healers interviewed had knowledge of *P. guajava *as an antidiarrhoeal agent. The antidiarrhoeal activity of *P. guajava *though widely reported has been mainly investigated with respect to its antimicrobial action and/or its effect on physiological diarrhoea in animal models [17]. Thus its mechanism(s) of action in infectious diarrhoea is largely unknown. To the best of our knowledge there have been no studies till date on the effect of the leaf extract of *P. guajava *on the pathogenesis of infectious diarrhoea.

Hence, in the present study the crude aqueous extract of the leaves of *P. guajava *was studied for its effect on various virulence parameters of infectious diarrhoea viz., colonization on intestinal epithelial cells and production and action of enterotoxins. The activity of the crude extract was compared with that of quercetin to ascertain its role in the observed biological activity. Quercetin is a major flavonoid present in *P. guajava *leaves and has been previously reported to have antidiarrhoeal activity [17]. Quercetin has also been used as a marker for standardization of crude aqueous leaf extract [19].

## Methods

### *Plant material, preparation of decoction, quercetin*

The leaves of *P. guajava*, variety Sardar which is amongst the 5 common varieties found in India, were collected from Parinche valley, Maharashtra, India. A voucher specimen of the plant material has been deposited at the Botanical Survey of India, Western circle, Pune, India with herbarium number 124672. The leaves were shade dried, powdered, and stored at 4°C. All experiments were performed with the same plant material.

As described in the Ayurvedic texts [20], the decoction was prepared by boiling 1 g of the plant material in 16 ml double distilled water till the volume reduced to 4 ml. To replicate field conditions a fresh decoction was prepared every time. The decoction was centrifuged and filtered through a membrane of 0.22 μm pore size before use. For each experiment, 0.1% (1:1000 dilution), 1% (1:100 dilution), 5% (1:20 dilution), and 10% (1:10 dilution) (v/v) concentrations of the decoction and corresponding dilutions of quercetin in appropriate media were used. The amount of quercetin present in the decoction was estimated by a method as described under the section of phytochemical analysis.

### *Media, reagents, plastic ware and instrumentation*

The bacterial media was purchased from HiMedia laboratory, Mumbai, India. Dulbecco's modified Eagle medium (DMEM) and fetal calf serum (FCS) were procured from GibcoBRL, UK. Quercetin, anti-cholera toxin and bovine serum albumin were purchased from Sigma, USA. Peroxidase labeled swine anti-rabbit immunoglobulin was from Dako, Denmark. All chemicals were from SD Fine Chemicals, Mumbai. Gallic acid was kindly provided by Dr. K. S. Laddha, Institute of Chemical Technology, Mumbai, India. Lactulose was a product of Intas Pharmaceuticals, Ahmedabad, India. The 24- and 96-well tissue culture plates and the 96-well ELISA plates were purchased from Nunclon, Denmark, the 55 mm diameter tissue culture plates were obtained from Tarsons, Kolkata, India and the ELISA plate reader was purchased from Labsystems, Finland. The pre-coated Silica gel plates for high performance thin layer chromatography, G60 F254 TLC, were obtained from Merck, Germany.

### *Cell culture*

The human laryngeal epithelial cell line, HEp-2 was obtained from the National Centre for Cell Sciences, Pune. The cell line was maintained in DMEM supplemented with 10% FCS, at 37°C in a 5% CO_2 _atmosphere. The cells were maintained in logarithmic growth by passage every 3-4 days.

### *Microorganisms used*

Six bacteria viz., enteropathogenic *Escherichia coli *(EPEC) strain B170, serotype 0111:NH, enterotoxigenic *E. coli *(ETEC) strains B831-2, serotype unknown (heat labile toxin producer) and TX1, serotype 078:H12 (heat stable toxin producer) (all strains obtained from Centre for Disease Control, Atlanta, USA), enteroinvasive *E. coli *(EIEC) strain E134, serotype 0136:H- (kindly provided by Dr. J. Nataro, Veterans Affairs Medical Centre, Maryland, USA); *Vibrio cholerae *C6709 El Tor Inaba, serotype 01 (kindly provided by Dr. S. Calderwood, Massachusetts General Hospital, Boston, USA) and *Shigella flexneri *M9OT, serotype 5 (kindly provided by Dr. P. Sansonetti, Institut Pasteur, France) were used for the present study.

### *Phytochemical analysis*

The decoction was qualitatively assayed for presence of carbohydrates, glycosides, proteins, phytosterols, saponins, flavonoids, alkaloids and tannins [21].

High performance thin layer chromatography (HPTLC) fingerprinting of the methanol soluble fraction of the decoction was carried out on pre-coated Silica gel G60 F254 TLC plates with quercetin as a standard. The plates were developed in solvent system consisting of Dichloromethane:Ethyl acetate:Methanol:Acetic acid (8:4:2:0.1). The chromatogram was derivatized with ferric chloride which is specific for quercetin and scanned using TLC Scanner 3 (CAMAG) in visible light. The amount of quercetin in *P. guajava *decoction was estimated by preparing a standard graph by running different concentrations of standard quercetin and measuring the intensity of the spots by using the software Multigauge version 3.0 (Fugifilm). The intensity of the corresponding spot obtained with the decoction (hydrolysed using 1N Hydrochloric acid) was measured and extrapolated onto the standard graph to estimate the amount of total quercetin in the decoction. The acid hydrolysed decoction was used so as to obtain the conjugated quercetin in free form.

The concentration of quercetin in the decoction was also confirmed by high performance liquid chromatography (HPLC) using HPLC system from Dionex Corp., USA. The column used was 250 mm × 4.6 mm 15 μC_18 _120 Å Acclaim. 20 μl of sample diluted 1:1 with Water: Acetonitrile:Phospohoric Acid (50:50:0.1) was run at a flow rate of 1 ml/min with Water: Acetonitrile:Phospohoric Acid as the mobile phase at the ratios of 90:10:0.1 and 10:90:0.1 in Phase A and Phase B respectively. The column oven temperature was adjusted to 30°C and the readings were taken at a wavelength of 210 nm.

#### *Antibacterial activity*

The antibacterial activity was determined by a microtitre plate based assay [22]. The bacterial strains were incubated with the decoction/quercetin in nutrient broth and the optical density was measured after 24 h as a measure of growth. Ofloxacin (1 μg/ml) was used as the antibiotic control. Three independent experiments were carried out. In each experiment, triplicate wells were set up for control as well as for each dilution of the decoction/quercetin.

### *Effect on bacterial colonization*

#### *Effect on adherence*

The effect on the adherence of *E. coli *B170 to epithelial cells was assayed by a method described by Cravioto *et al*. [23]. Briefly, a 48 h culture of HEp-2 cells on glass coverslips was infected with a log phase culture (5 × 10^7^/ml) of the bacteria in DMEM and incubated for 3 h. Non-adherent bacteria were washed off, the coverslips fixed in 10% formaldehyde and stained with toluidine blue stain (0.1% w/v). HEp-2 cells having typical EPEC microcolonies [24] were counted under light microscope. Three independent experiments were carried out. In each experiment, duplicate coverslips were set up for control as well as for each dilution of the decoction/quercetin.

The effect of the decoction/quercetin on adherence of *E. coli *B170 to HEp-2 cells was compared with that of lactulose, a prebiotic oligosaccharide, known to inhibit adherence of EPEC to tissue culture cells [25].

#### *Effect on invasion*

The effect on invasion of *E. coli *E134 and *S. flexneri *to epithelial cells was studied by a method described by Vesikari *et al*. [26]. Briefly, a 48 h culture of HEp-2 cells grown in a 24-well tissue culture plate was infected with log phase culture (10^8^/ml) of the bacteria in DMEM and incubated for 2 h. The extracellular bacteria were then washed off and the culture was further incubated with DMEM containing gentamycin (100 μg/ml) for 3 h. The medium containing gentamycin was washed off and the epithelial cells were then lysed using chilled distilled water and the released bacteria were enumerated by plating on nutrient agar. Three independent experiments were carried out. In each experiment, duplicate wells were set up for control as well as for each dilution of the decoction/quercetin.

The effect of the decoction/quercetin on invasion of *S. flexneri *was also compared with that of lactulose, as it has been used for the treatment of shigellosis and inflammatory bowel disease [27]. Since the mechanism of invasion of both EIEC and *S. flexneri *is almost identical [28], the effect of the decoction on invasion of *E. coli *E134 was also compared with that of lactulose.

Two different protocols were performed for both the adherence and the invasion assays to understand whether the bacterial adherence and invasion respectively were affected by the effect of the decoction/quercetin on the epithelial cells or through competitive inhibition. The HEp-2 cells were incubated in absence (control) and presence of different dilutions of the decoction/quercetin either for 18-20 h prior to infection (pre-incubation) or simultaneously with infection (competitive inhibition) respectively.

### *Effect on bacterial enterotoxins*

#### *Effect on E. coli heat labile toxin (LT) and cholera toxin (CT)*

LT, which is localized in the cell membrane of *E. coli *B831-2 was obtained by lysing the bacterial cells with polymyxin B sulphate (1 mg/ml) whereas CT, which is released extracellularly, was obtained as a culture supernatant of *V. cholerae*. LT and CT were assayed by the ganglioside monosialic acid enzyme linked immunosorbent assay (GM1-ELISA) [29]. Briefly, the toxins were added to ELISA plates pre-coated with 1.5 μmol/ml of GM1. Anticholera toxin (1:300) and peroxidase labeled swine anti-rabbit immunoglobulin (1:200) were used as primary and secondary antibodies respectively. Orthophenylene diamine was used as the substrate. The intensity of the color thus developed was read at 492 nm in an ELISA plate reader.

The effect of the decoction/quercetin on production of these toxins was compared with that of 2-mercaptoethanol since thiols such as 2-mercaptoethanol, L-cysteine monohydrochloride and sodium thioglycolate have been reported to inhibit production of LT, CT and ST [30,31]. Similarly, their effect on binding of these toxins to GM1 was compared with that of gallic acid, a polyphenol, which has been reported to block the binding of LT to GM1 [32]. As LT and CT are antigenically closely related [33], the effect of the decoction on the binding of CT was also compared to that of gallic acid.

To study the effect on production of the toxins, the respective bacteria were grown in Casein hydrolysate yeast extract (CAYE) in absence (control) and presence of different dilutions of the decoction/quercetin and the toxins produced were assayed by GM1-ELISA. To study the effect on the binding of the toxins to GM1, the toxins obtained by growing the respective bacteria in CAYE were added to the assay system in absence (control) and presence of the decoction/quercetin. Three independent experiments were carried out. In each experiment, triplicate wells were set up for control as well as for each dilution of the decoction/quercetin.

#### *Effect on stable toxin (ST)*

ST, which is released extracellularly was obtained as the culture supernatant of *E. coli *TX1 and assayed by the method originally described by Gianella [34]. Briefly, the toxin was inoculated intragastrically in 2-3 day old Swiss White suckling mice. Following an incubation of 3 h at room temperature, the animals were sacrificed and the ratio of gut weight to that of the remaining carcass weight was calculated. Ratio of ≥ 0.083 was considered as positive for fluid accumulation.

To study the effect on production of ST, the bacterium was grown in CAYE in absence (control) and presence of different dilutions of the decoction/quercetin and the toxin produced was assayed. To study the effect on the action of ST, the toxin obtained by growing the bacterium in CAYE was intragastrically injected in absence (control) and presence of different dilutions of the decoction/quercetin. CT was used as a negative control. Three independent experiments were carried out. In each experiment, three animals were inoculated for control as well as each dilution of the decoction/quercetin.

The Institutional Ethics Committee and the Committee for the Purpose of Control and Supervision of Experiments on Animals (CPCSEA) cleared the use of animals in the study. The Foundation for Medical Research (FMR) is registered with CPCSEA (registration No. 424/01/a/CPCSEA, June 20th, 2001).

### *Statistical analysis and presentation of data*

The results for each assay have been expressed as the mean ± standard error of the percentage values from three independent experiments. The percentage in each experiment was calculated using the formula {(C or T)/C} × 100, where C is the mean value of the duplicate/triplicate readings of the control group and T is mean value of the duplicate/triplicate readings of the test (dilutions of the decoction/quercetin) groups. Hence, the value of control is 100% and the values of the test groups have been represented as percentages relative to control.

Data were analyzed by analysis of variance (ANOVA) and Dunnett's post test. *P *≤ 0.05 was considered to be statistically significant. The EC_50 _values, wherever applicable, were calculated by nonlinear regression analysis using the equation for a sigmoid concentration-response curve. All statistical analyses were performed using the software Prism 4.0 (GraphPad, Inc.).

## Results

### *Phytochemistry*

The percent yield of the decoction with respect to the starting dried plant material was 10.8% ± 0.5% (w/w). The dry weight of the decoction was 27.0 mg/ml ± 1.25 mg/ml. Thus the concentrations of the different dilutions used in the biological assays were 0.027 mg/ml ± 0.001 mg/ml (0.1%), 0.270 mg/ml ± 0.013 mg/ml (1%), 1.850 mg/ml ± 0.063 mg/ml (5%) and 2.700 mg/ml ± 0.125 mg/ml (10%).

The amount of total quercetin was estimated to be 2 mg per gram of the dried leaves of *P. guajava*. Quercetin was used at concentrations of 2 μg/ml, 20 μg/ml, 100 μg/ml and 200 μg/ml which corresponded to the amount of quercetin present in the different concentrations of the decoction used for the study.

The classes of compounds present in the decoction were carbohydrate, reducing sugars, proteins, saponins, flavonoids, tannins, and traces of alkaloids. Phytosterols and glycosides were absent.

**Figure 1:**
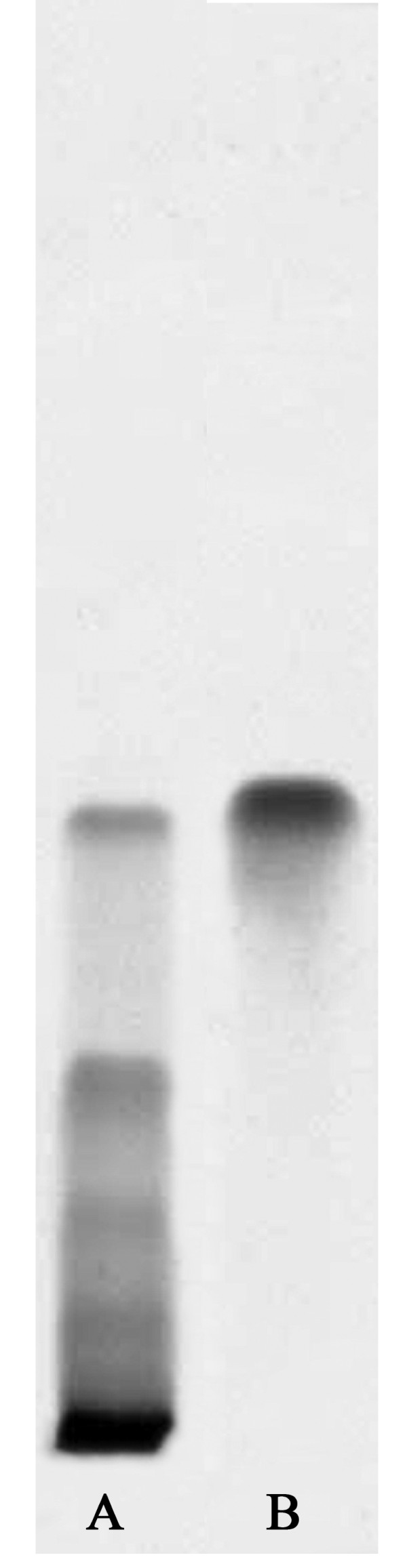
**HPTLC profile of the decoction of *P. guajava***. (A) Methanol fraction of the decoction. (B) Quercetin.

The HPTLC profile of the methanol soluble fraction of the decoction is presented in Figure 1. The fingerprint shows presence of quercetin as one of the constituents of the decoction (R_f _value 0.58). The HPLC profile has been provided in additional file 1.

### *Antibacterial activity*

**Figure 2:**
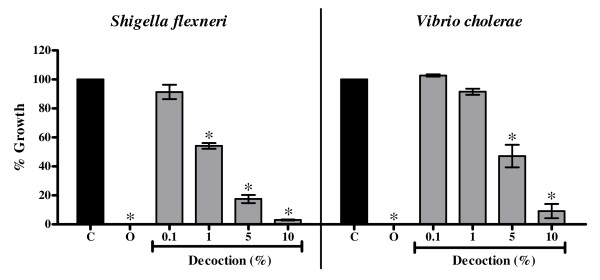
**Antibacterial activity of decoction of *P. guajava *against *S. flexneri *and *V. cholerae***. C: Control, O: Ofloxacin (1 μg/ml). Values represent mean ± standard error (n = 3) of percentage bacterial growth relative to control (100%); * *P *< 0.05.

The decoction inhibited the growth of two of the six bacterial strains tested viz., *S. flexneri *(EC_50 _value 0.98% ± 0.2%) and *V. cholerae *(EC_50 _value 2.88% ± 0.36%) (Figure 2) whereas there was no effect of quercetin at any of the concentrations tested on the growth of all six strains tested (data not shown). Ofloxacin at a concentration of 1 μg/ml completely inhibited the growth of all the bacterial strains.

### *Effect on bacterial colonization*

#### *Adherence of E. coli B170*

In the competitive protocol, 15 mg/ml of lactulose was used as a positive control whereas in the pre-incubation protocol 2.5 mg/ml of lactulose was used since it was toxic to HEp-2 cells even at 5 mg/ml when incubated overnight with the cells. Below this concentration lactulose had no effect on the adherence.

**Figure 3:**
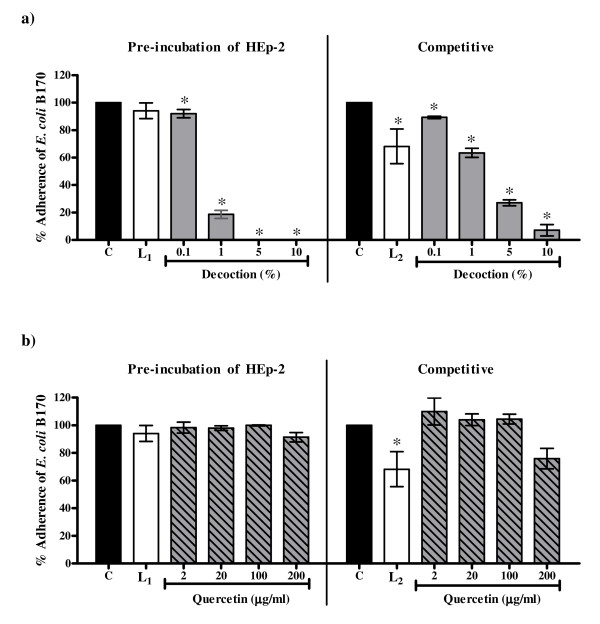
**Effect on adherence of *E. coli *B170 to the HEp-2 cells in the pre-incubation and the competitive protocols with a) the decoction of *P. guajava*; b) quercetin**. C: Control, bacterial adherence to HEp-2 cells in medium alone; L_1_: Bacterial adherence to HEp-2 cells when pre-incubated in medium with 2.5 mg/ml lactulose; L_2_: Bacterial adherence to HEp-2 cells in medium with 15 mg/ml lactulose in the competitive protocol. Values represent mean ± standard error (n = 3) of percentage adherence relative to control (100%); * *P *< 0.05.

As compared to lactulose the decoction showed a greater decrease in the adherence of *E. coli *B170 to HEp-2 cells in both the pre-incubation (EC_50 _value 0.37% ± 0.05%) and the competitive protocols (EC_50 _value 1.27% ± 0.18%) (Figure 3a). However, quercetin had no effect on bacterial adherence (Figure 3b). This suggests that the crude decoction contains active components(s), other than quercetin, which may be responsible for the decrease in adherence. Though the decoction reduced the adherence of *E. coli *B170 in both the protocols, the extent of decrease was greater in the pre-incubation protocol. The decrease in adherence in the competitive protocol cannot be attributed to antibacterial activity since the decoction was not cidal to *E. coli *B170. Thus it is likely that the components of the decoction act either on HEp-2 cell metabolism or block the receptors on the cell surface specific for bacterial adhesins thereby preventing the attachment of the bacteria.

#### *Invasion of E. coli E134 and S. flexneri to HEp-2 cells*

As compared to lactulose (2.5 mg/ml) the crude decoction as well as quercetin showed greater inhibition of the invasion of *E. coli *E134 and *S. flexneri *in both the pre-incubation and the competitive protocols. The EC_50 _values for inhibition of invasion of *E. coli *E134 with the *P. guajava *decoction in the pre-incubation (0.23% ± 0.02%) and competitive (0.25% ± 0.01%) protocols were significantly lower as compared to that of the EC_50 _values with quercetin (39.36 μg/ml ± 3.27 μg/ml and 55.87 μg/ml ± 13.14 μg/ml respectively which correspond to 1.97% ± 0.16% and 2.79% ± 0.66% of the crude decoction). Similarly, the EC_50 _values for inhibition of invasion of *S. flexneri *in the competitive (0.19% ± 0.06%) protocol with *P. guajava *decoction was significantly lower as compared to that of the EC_50 _value with quercetin (41.6 μg/ml ± 9.11 μg/ml corresponding to 2.08% ± 0.46% of the crude decoction ) whereas in the pre-incubation protocol the EC_50 _value with the decoction (0.19% ± 0.06%) was comparable with that of the EC_50 _value with quercetin (0.81 μg/ml ± 1.03 μg/ml corresponding to 0.04% ± 0.05% of the crude decoction).

Since the extent of decrease in invasion was similar in both the protocols it suggests that both the decoction and quercetin probably affect HEp-2 cell metabolism. As discussed earlier, since the decoction and quercetin had no effect on viability of *E. coli *E134, the reduction in invasion of this bacterium in the competitive protocol cannot be attributed to antibacterial activity. Likewise though the decoction was cidal to *S. flexneri*, the decrease in invasion of this strain in the competitive protocol even at 1% concentration (at which it had no bactericidal activity) indicates that the effect of the decoction on invasion of this bacterium was not only due to direct killing of the bacteria.

**Figure 4:**
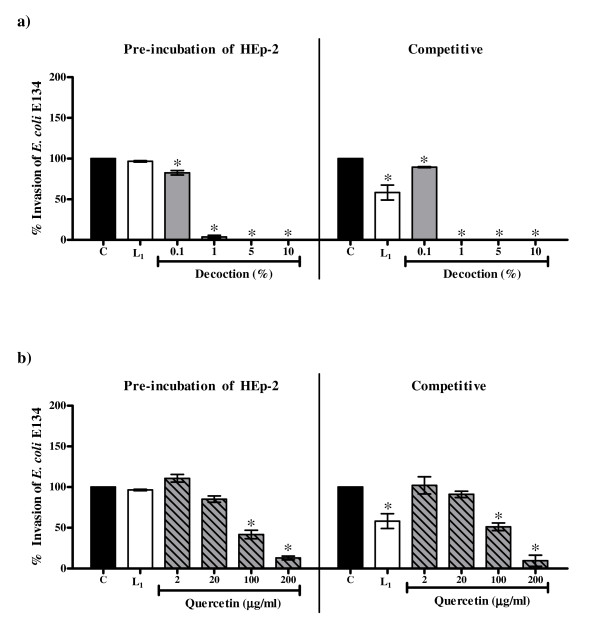
**Effect on invasion of *E. coli *E134 to the HEp-2 cells in the pre-incubation and the competitive protocols with a) the decoction of *P. guajava*; b) quercetin**. C: Control, bacterial invasion to HEp-2 cells in medium alone; L_1_: Bacterial invasion to HEp-2 cells in medium with 2.5 mg/ml lactulose. Values represent mean ± standard error (n = 3) of percentage invasion relative to control (100%); * *P *< 0.05.

**Figure 5:**
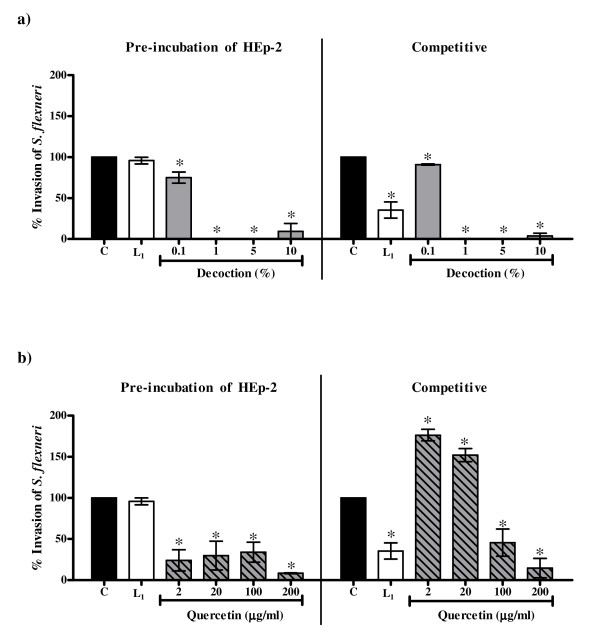
**Effect on invasion of *S. flexneri *to the HEp-2 cells in the pre-incubation and the competitive protocols with a) the decoction of *P. guajava*; b) quercetin**. C: Control, bacterial invasion to HEp-2 cells in medium alone; L_1_: Bacterial invasion to HEp-2 cells in medium with 2.5 mg/ml lactulose. Values represent mean ± standard error (n = 3) of percentage invasion relative to control (100%); * *P *< 0.05.

It may be noted that the extent of decrease with quercetin was less than the decoction (Figure 4 and Figure 5). In fact 2 μg and 20 μg of quercetin corresponding to 0.1% and 1% respectively of the decoction showed no significant reduction in invasion of *E. coli *E134 (Figure 4b) in the two protocols used and surprisingly increased the invasion of *S. flexneri *in the competitive protocol (Figure 5b).

### *Effect on bacterial enterotoxins*

#### *LT*

**Figure 6:**
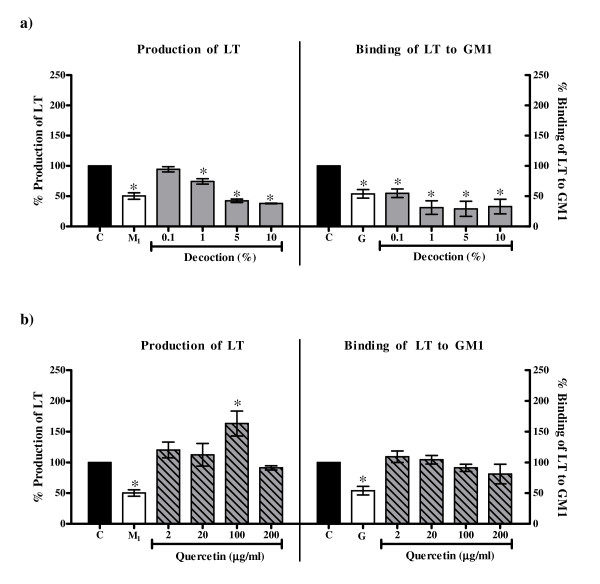
**Effect on production of LT and its binding to GM1 with a) the decoction of *P. guajava*; b) quercetin**. C: Control, LT in medium alone; M_1_: LT in medium with 5 mM 2-mercaptoethanol; G: Toxin in medium with 50 mM gallic acid. Values represent mean ± standard error (n = 3) of percentage production/binding relative to control (100%); * *P *< 0.05.

The decrease in the production of LT by *E. coli *B831-2 (EC_50 _value 1.03% ± 0.32%) and its binding to GM1 (EC_50 _value 0.06% ± 0.02%) by the decoction was comparable to that of 2-mercaptoethanol (5 mM) and gallic acid (50 mM) respectively (Figure 6a). Quercetin, on the other hand, increased the production of LT at concentration of 100 μg/ml (Figure 6b) but did not affect the binding to GM1. These results suggest that the decoction, in addition to quercetin, contains compounds which nullify the enhancing effect of quercetin resulting in an overall decrease in the production of LT. The effect of the decoction and quercetin on LT production by *E. coli *B831-2 is indicative of their effect on bacterial metabolism.

#### *CT*

**Figure 7:**
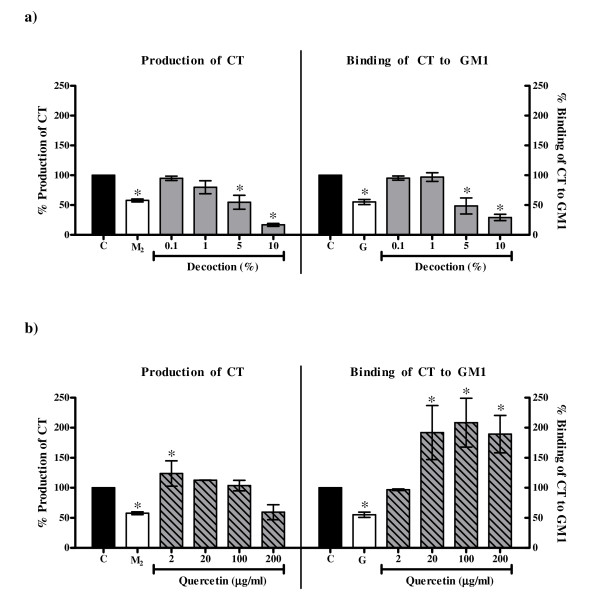
**Effect on production of CT and its binding to GM1 with a) the decoction of *P. guajava*; b) quercetin**. C: Control, toxin in medium alone; M_2_: CT in medium with 1 mM 2-mercaptoethanol; G: Toxin in medium with 50 mM gallic acid. Values represent mean ± standard error (n = 3) of percentage production/binding relative to control (100%); * *P *< 0.05.

The decoction showed an overall greater decrease in production of CT (EC_50 _value 2.69% ± 1.18%) and its binding to GM1 (EC_50 _value 2.51% ± 1.07%) (Figure 7a) as compared to 2-mercaptoethanol (1 mM) and gallic acid (50 mM) respectively. Quercetin did not inhibit the production of CT but increased the binding of CT to GM1 (Figure 7b). As LT and CT are largely antigenically related [33], the difference in the pattern of binding of LT and CT to GM1 in presence of quercetin is suggestive of the additional affinity of quercetin to determinants specific to CT.

It may be noted that the decrease in production of CT (unlike LT) by the decoction may be due to the cidal action of the decoction against *V. cholerae *at 5% and 10% concentrations. Thus analogous to the observations made in the colonization assays, these results are also suggestive of the presence of other 'active components' in the decoction in addition to quercetin.

#### *ST*

The production and action of ST was unaffected by the decoction as well as quercetin (data not shown). This suggests the selective action of the decoction and quercetin against LT and CT.

## Discussion

The pathogenesis of infectious diarrhoea has been widely studied. Enteric pathogens have evolved a remarkable array of virulence traits that enable them to colonize the intestinal tract. These organisms colonize and disrupt intestinal function to cause malabsorption or diarrhoea by mechanisms that involve microbial adherence and localized effacement of the epithelium, production of enterotoxin(s) and direct epithelial cell invasion [35]. Adherence is a means of colonizing the appropriate ecological niche, which enables the organism to resist being swept away by mucosal secretions, with subsequent proliferation and colonization in the gut and may be followed by enterotoxin production or invasion [36]. Thus, these are important parameters in the pathogenesis of diarrhoea and can be used for understanding the varied mechanism(s) of action of antidiarrhoeal medicinal plants. These parameters have been employed by us [37-42] to assess the antidiarrhoeal activity of selected medicinal plants. In the present study the antidiarrhoeal action of *P. guajava *leaves has been assessed using a similar approach. It may be noted that our studies deviate from a number of other studies on antidiarrhoeal activity of medicinal plants which are mostly restricted to intestinal motility and antimicrobial activity [43-53] and thus overlook the pathogenesis of infectious diarrhoea.

*P. guajava *is not only popular as a tropical plant which is widely consumed and processed, but also as an important food crop with several commercial applications and medicinal properties [17]. It is considered as a native to Mexico and extends throughout the South America, Europe, Africa and Asia. It grows in all the tropical and subtropical areas of the world and adapts to different climatic conditions. Recent ethnopharmacological studies have shown various medicinal uses for *P. guajava *apart from its traditional use in gastrointestinal disorders, in dermatologic conditions, as an anti-inflammatory, for diabetes, hypertension, caries, wounds, pain relief, and reducing fever [17]. Although all parts of this plant are known to possess several medicinal properties, the leaves and fruits, in particular, have been widely studied and attributed with various pharmacological properties.

The restriction of crude extract for the study was intentional and based on the belief that it would represent the nearest possible form to traditional preparations that can be used by the locals. This has also been emphasized recently by Rosales-Reyes *et al*. [54]. Studies reporting the antidiarrhoeal activity of the aqueous extracts of *P. guajava *leaves both in animal models as well as in clinical trials [17] further strengthen the use of decoction in the present study. Additionally, the safety of the crude aqueous extract of *P. guajava *leaves has been well demonstrated. A LD_50 _value of 1534 ± 69 mg/kg (IP) in mice was obtained in a recent study carried out by Ojewole *et al*. [55]. Oral administration of 100-500 mg/Kg body weight of the aqueous extract of *P. guajava *leaves has been shown to have no significant harmful effect in Winstar rats after 72 h [56]. The water extract of *P. guajava *leaves was found to be effective in inactivating the mutagenic effects of agents such as 4-nitro-o-phenylenediamine, sodium azide and 2-aminofluorene on *S. typhimurium *in Ames assay [57]. *In vivo *and *in vitro *cytotoxicity and mutagenicity tests of aqueous extract of the leaves in Winstar rat bone marrow cells and human peripheral blood lymphocytes respectively did not show any statistically significant alterations in either the cell cycle or the number of chromosome alterations [58].

Quercetin one of the most abundant flavonoids and biologically active constituent present in *P. guajava *leaves [17] has also been reported to have many beneficial effects on human health, including cardiovascular protection, anti-cancer activity, anti-ulcer effects, anti-allergy activity, cataract prevention, antiviral activity, and anti-inflammatory effects [59]. Quercetin has been reported to be antispasmodic [19,60,61] and also to inhibit gastrointestinal release of acetylcholine [62]. Hence, in this study quercetin was also screened along with the crude decoction of *P. guajava *leaves to elucidate its contribution towards the efficacy of the decoction in the bioassays employed herein.

Through the results of the present study, the antidiarrhoeal activity of the crude aqueous decoction of *P. guajava *is evident even in the absence of marked antibacterial action. The decrease in bacterial colonization of HEp-2 cells was seen in both the protocols (pre-incubation and competitive) used. This indicates that *P. guajava *affects HEp-2 cell metabolism. Similarly, the decrease in production of LT without arresting the growth of *E. coli *B831-2 is suggestive of *P. guajava *also being able to affect bacterial metabolism.

On the other hand, quercetin had no effect on adherence of *E. coli *B170 to HEp-2 cells in both the protocols but affected the invasion of both *E. coli *E134 as well as *S. flexneri *in the two protocols used at 100 μg/ml and 200 μg/ml. Thus like the crude decoction, quercetin seems to affect HEp-2 cell metabolism. However as quercetin did not decrease bacterial adherence it is probable that unlike the crude decoction it does not block the cell surface receptors of HEp-2 cells. It is also possible that the modulation of HEp-2 cell metabolism by quercetin may be at a post adherence stage. Interestingly, the decrease in invasion of *S. flexneri *as compared to *E. coli *E134 in the pre-incubation protocol was noted at the lower concentrations (2 μg/ml and 20 μg/ml) of quercetin (Figure 5b). Since the mechanism of invasion of both EIEC and *S. flexneri *is almost identical [28], at present it is difficult to explain this observation. Though quercetin increased the production of LT, like the crude decoction it seems to affect bacterial metabolism as well.

Thus the comparative results of the crude decoction of *P. guajava *leaves and quercetin illustrate that quercetin alone is not responsible for all the observed biological activities of the crude leaf decoction. It has been reported previously that constituents such as tannins, flavonoids and saponins in general have antidiarrhoeal activity [43,47-50,53,63-66] which have been attributed to their antimotility and antisecretory effects [45,50,51,64] and antimicrobial action [47,48,52]. As tannins, flavonoids and saponins were found to be present in the crude decoction of *P. guajava *leaves, these constituents may be responsible for the observed activities in the present study. Therefore, it is speculated that the efficacy of crude extract may be due to the interplay between the different active constituents that may be present in the extract leading to better activity and/or decrease in potential toxicity of some individual constituents. Alternatively, the individual action of different constituents present in the extract may collectively contribute to the efficacy of the extract. This is illustrated by observations obtained from the present study wherein the extent of decrease of bacterial invasion with crude extract was greater than that with quercetin alone and the ability of the crude decoction (and not quercetin) to inhibit adherence and LT/CT. The effect of the interplay of different constituents is also evident from the observation that the enhancement of invasion of *S. flexneri *to HEp-2 cells in the presence of 2 μg/ml and 20 μg/ml of quercetin (Figure 5b) was abrogated in the crude decoction (Figure 5a). A similar observation has been made by Mavar-Manga *et al*. [67] who demonstrated that different constituents of crude extracts act on different mechanisms.

## Conclusion

The present study demonstrates the usefulness of *P. guajava *leaves in different forms of infectious diarrhoea. In the absence of marked antibacterial activity, the inhibition of the production and the action of *E. coli *heat labile toxin and cholera toxin and reduction of bacterial colonization (both adherence and invasion) to epithelial cells are the probable modes of action of *P. guajava *leaves. The study also showed that the crude decoction of *P. guajava *leaves contains components other than quercetin which contribute to its antidiarrhoeal action.

The mechanisms of antidiarrhoeal activity of *P. guajava *leaves as proposed above along with other reported mechanism(s) of action viz., antimicrobial activity, antispasmodic activity, inhibition of increased watery secretion and inhibition of acetylcholine release [17], strengthen the ethnomedical usage of *P. guajava *leaf in different forms of diarrhoea. Hence this study besides providing newer insights into the varied possible antidiarrhoeal mechanisms of *P. guajava *leaves also adds credence to the existing traditional knowledge about this plant and justifies its continued use globally.

## Competing interests

The authors declare that they have no competing interests.

## Authors' contributions

TB and NA were responsible for the study. PD and BS carried out the laboratory studies, analyzed the data and were involved in the preparation of the manuscript. PT collected the plant material, authenticated it and obtained the voucher specimen number. AN guided the phytochemical studies. All the authors except Late Dr. Noshir Antia have read and approved the final version of the manuscript.

## Pre-publication history

The pre-publication history for this paper can be accessed here:

http://www.biomedcentral.com/1472-6882/10/33/prepub

## Supplementary Material

Additional file 1 **HPLC profile of the decoction of *P. guajava***. HPLC profile of the acid hydrolysed decoction of *P. guajava *shows the presence of the standard reference compound quercetin. Click here for file

## References

[B1] World Health Organization World Health Report Geneva: WHO 2004 120 125 http://www.who.int/whr/2004/annex/topic/en/annex_2_en.pdf

[B2] Murray CJL Lopez AD Alternative projections of mortality and disability by cause 1990-2020: Global burden of disease study Lancet 1997 349 1498 1504 10.1016/S0140-6736(96)07492-2 9167458

[B3] Casburn-Jones AC Farthing MJ Management of infectious diarrhoea Gut 2004 53 296 305 10.1136/gut.2003.022103 14724167 PMC1774945

[B4] Meyrowitsch DW Bygbjerg IbC Global burden of disease - a race against time Dan Med Bull 2007 54 32 34 17349218

[B5] Guerrant RL Bobak DA Bacterial and protozoal gastroenteritis N Engl J Med 1991 325 327 40 2057037 10.1056/NEJM199108013250506

[B6] Martines J Phillips M Feachem RGA Janson DT, Measham A Diarrhoeal Diseases Disease control priorities in developing countries 1993 UK: Oxford University Press 91 116

[B7] Bhattacharya SK Therapeutic methods for diarrhoea in children World J Gasteroenterol 2000 6 497 500 http://www.wjgnet.com/1007-9327/6/497.pdf 10.3748/wjg.v6.i4.497 PMC4723546 11819636

[B8] Dham SK Raghunath D, Nayak R Treatment of diarrheal diseases Diarrhoeal diseases: Current status, research trends and field studies 2003 New Delhi: Tata McGraw-Hill Publishing Company Limited 245 254

[B9] Cohen D Orr N Haim M Ashkenazi S Safety and immunogenicity of two different lots of the oral, killed enterotoxigenic *Escherichia coli *- cholera toxin B subunit vaccine in Israeli young adults Infect Immun 2000 68 4492 497 10.1128/IAI.68.8.4492-4497.2000 10899847 PMC98356

[B10] Ghose AC Adherence and colonization properties of *Vibrio cholerae *and diarrhoeagenic *Escherichia coli* Indian J Med Res 1996 104 38 51 8783506

[B11] Klee SR Tzschaschel BD Falt I Kaenell A Linberg AA Timmis KN Guzman CA Construction and characterization of a live attenuated vaccine candidate against *Shigella dysenteriae *type1 Infect Immun 1997 65 2112 2218 9169740 10.1128/iai.65.6.2112-2118.1997 PMC175292

[B12] Lagos R Fasano A Wasserman SS Prado V Martin OS Abrego P Losonsky GA Alegria S Levine MM Effect of small bowel bacterial overgrowth on the immunogenicity of single-dose live cholera vaccine CVD 103-HgR J Inf Dis 1999 180 1709 1712 10.1086/315051 10515838

[B13] Ryan ET Calderwood SB Cholera vaccines Clin Infect Dis 2000 31 561 565 10.1086/313951 10987721

[B14] Calain P Chaine JP Johnson E Hawley ML O'Leary MJ Oshitani H Chaignat CL Can oral cholera vaccination play a role in controlling a cholera outbreak? Vaccine 2004 355 2444 2451 10.1016/j.vaccine.2003.11.070 15193408

[B15] Dennehy PH Rotavirus vaccines: an update Curr Opin Pediatr 2005 17 88 92 10.1097/01.mop.0000147907.30720.04 15659970

[B16] Gutierrez SP Sanchez MAZ Gonzalez CP Garcia LA Antidiarrhoeal activity of different plants used in traditional medicine Afr J Biotech 2007 6 2988 2994 http://www.academicjournals.org/AJB/abstracts/abs2007/28Dec/Guti%C3%A9rrez%20et%20al.htm

[B17] Gutiérrez RM Mitchell S Solis RV *Psidium guajava*: a review of its traditional uses, phytochemistry and pharmacology J Ethnopharmacol 2008 117 1 27 10.1016/j.jep.2008.01.025 18353572

[B18] Tetali P Waghchaure C Daswani PG Antia NH Birdi TJ Ethnobotanical survey of antidiarrhoeal plants of Parinche valley, Pune district, Maharashtra, India J Ethnopharmacol 2009 123 229 36 10.1016/j.jep.2009.03.013 19429366

[B19] Lozoya X Reyes-Morales H Chávez-Soto MA Martínez-García Mdel C Soto-Gnzález Y Doubova SV Intestinal anti-spasmodic effect of a phytodrug of *Psidium guajava *folia in the treatment of acute diarrheic disease J Ethnopharmacol 2002 83 19 24 10.1016/S0378-8741(02)00185-X 12413703

[B20] Thakkur CG Introduction to ayurveda: Basic Indian medicine 1976 2 Jamnagar: Gulakunverba Ayurvedic Society

[B21] Kokate CK Purohit AP Gokhale SB Pharmacognosy 1990 1 Pune: Nirali Prakashan

[B22] Sarker SD Nahar L Kumarasamy Y Microtitre plate-based antibacterial assay incorporating resazurin as an indicator of cell growth, and its application in the *in vitro *antibacterial screening of phytochemicals Methods 2007 42 321 324 10.1016/j.ymeth.2007.01.006 17560319 PMC1895922

[B23] Cravioto A Grass RJ Scotland SM Gran RJ Scotland SM Rowe B An adhesive factor found in strains of *E. coli *belonging to the traditional infantile enteropathogenic serotypes Curr Microbiol 1979 3 95 99 10.1007/BF02602439

[B24] Knutton S Baldini MM Kaper JB McNeish AS Role of plasmid-encoded adherence factors in adhesion of enteropathogenic *Escherichia coli *to HEp-2 cells Infect Immun 1987 55 78 85 2878887 10.1128/iai.55.1.78-85.1987 PMC260282

[B25] Shoaf K Mulvey GL Armstrong GD Hutkins RW Prebiotic galactooligosaccharides reduce adherence of enteropathogenic *Escherichia coli *to tissue culture cells Infect Immun 2006 74 6920 6928 10.1128/IAI.01030-06 16982832 PMC1698067

[B26] Vesikari T Bromisrska J Maki M Enhancement of invasiveness of *Yersinia enterocolitica *and *Escherichia coli *to HEp-2 cells by centrifugation Infect Immun 1982 36 834 836 7044978 10.1128/iai.36.2.834-836.1982 PMC351304

[B27] Liao W Cui XS Jin XY Florén CH Lactulose - a potential drug for the treatment of inflammatory bowel disease Med Hypotheses 1994 43 234 238 10.1016/0306-9877(94)90072-8 7838007

[B28] Nataro JP Kaper JB Diarrheagenic *Escherichia coli* Clin Microbiol Rev 1998 11 142 201 9457432 10.1128/cmr.11.1.142 PMC121379

[B29] Svennerholm A-M Wilkund G Rapid GM1-enzyme-linked immunosorbent assay with visual reading for identification of *Escherichia coli *heat-labile enterotoxins J Clin Microbiol 1983 17 596 600 6343419 10.1128/jcm.17.4.596-600.1983 PMC272699

[B30] Greenberg RN Dunn JA Guerrant RL Reduction of the secretory response to *Escherichia coli *heat-stable enterotoxin by thiol and disulfide compounds Infect Immun 1983 41 174 180 6134677 10.1128/iai.41.1.174-180.1983 PMC264759

[B31] Shimamura T Watanabe S Sasaki S Inhibition of cholera toxin production by thiols in *Vibrio cholerae* Infect Immun 1986 53 700 701 3744560 10.1128/iai.53.3.700-701.1986 PMC260851

[B32] Chen JC Ho TY Chang YS Wu SL Hsiang CY Anti-diarrheal effect of Galla Chinensis on the *Escherichia coli *heat-labile enterotoxin and ganglioside interaction J Ethnopharmacol 2006 103 385 391 10.1016/j.jep.2005.08.036 16213682

[B33] Ganguly NK Kaur T Mechanism of action of cholera toxin and other toxins Indian J Med Res 1996 104 28 37 8783505

[B34] Gianella RA Suckling mouse model for detection of heat stable *Escherichia coli *enterotoxin: characteristics of the model Infect Immun 1976 14 95 99 780280 10.1128/iai.14.1.95-99.1976 PMC420849

[B35] Guerrant RL Steiner TS Lima AAM Bobak DA How intestinal bacteria cause disease J Infect Dis 1999 179 S331 S337 10.1086/513845 10081504

[B36] Ashkenazi S Pickering LK Pathogenesis and diagnosis of bacterial diarrhoea Eur J Clin Microbiol Infect Dis 1989 8 203 206 10.1007/BF01965261 2496987

[B37] Brijesh S Daswani PG Tetali P Antia NH Birdi TJ Studies on *Dalbergia **sissoo *Roxb. leaves: Possible mechanism(s) of action in infectious diarrhoea Indian J Pharmacol 2006 38 120 124 10.4103/0253-7613.24618

[B38] Brijesh S Daswani PG Tetali P Rojatkar SR Antia NH Birdi TJ Studies on *Pongamia pinnata *(L.) Pierre leaves: Understanding the mechanism(s) of action in infectious diarrhoea J Zhejiang Univ Sci B 2006 7 665 674 10.1631/jzus.2006.B0665 16845722 PMC1533752

[B39] Brijesh S Daswani P Tetali P Antia N Birdi T Studies on the antidiarrhoeal activity of *Aegle marmelos *unripe fruit: Validating its traditional usage BMC Complement Altern Med 2009 9 47 10.1186/1472-6882-9-47 19930633 PMC2788518

[B40] Daswani PG Birdi TJ Antia NH Study of the action of *Cyperus rotundus *root decoction on the adherence and enterotoxin production of diarrhoeagenic *Escherichia coli* Indian J Pharmacol 2001 33 116 117 http://medind.nic.in/ibi/t01/i2/ibit01i2p116.pdf

[B41] Daswani PG Birdi TJ Antarkar DS Antia NH Investigation of the antidiarrhoeal activity of *Holarrhena antidysenterica* Indian J Pharm Sci 2002 64 164 167 http://www.ijpsonline.com/article.asp?issn=0250-474X;year=2002;volume=64;issue=2;spage=164;epage=167;aulast=Daswani;type=0

[B42] Daswani PG Brijesh S Tetali P Antia NH Birdi TJ Antidiarrhoeal activity of *Zingiber officinale *(Rosc.) Curr Sci 2010 98 2 222 229 http://www.ias.ac.in/currsci/25jan2010/222.pdf

[B43] Agbor GA Leopold T Jeanne NY The antidiarrhoeal activity of *Alchornea cordifolia *leaf extract Phytother Res 2004 18 11 873 876 10.1002/ptr.1446 15597302

[B44] Akah PA Aguwa CN Agu RU Studies on the antidiarrhoeal properties of *Pentaclethra macrophylla *leaf extracts Phytother Res 1999 13 292 295 10.1002/(SICI)1099-1573(199906)13:4<292::AID-PTR415>3.0.CO;2-N 10404533

[B45] Di Carlo G Autore G Izzo AA Maibline P Mascolo N Viola P Diurno MV Capasso F Inhibition of intestinal motility and secretion by flavonoids in mice and rats: Structure activity relationships J Pharm Pharmacol 1993 45 1054 1059 7908974 10.1111/j.2042-7158.1993.tb07180.x

[B46] Kambu K Tona L Luki N Cimanga K Uvoya A Antibacterial activity of extracts from plants used in preparations as antidiarrhoeal at Kinshasa, Zaire Ann Pharm Fr 1990 48 255 263 2088161

[B47] Lutterodt GD Ismail A Basheer RH Mohd Baharuddin H Antimicrobial effects of *Psidium guajava *extract as one mechanism of its antidiarrhoeal action Malaysian J Med Sci 1999 6 17 20 http://ernd.usm.my/journal/journal/ANTIMICROBIAL%20EFFECTS%20OF%20PSIDIUM%20GUAJAVA%20EXTRACT%20AS%20ONE%20MECHANISM%20OF%20ITS%20ANTIDIARRHOEAL%20ACTION.pdf PMC3329747 22589684

[B48] Miranda D Pereira L Sirsat SM Antarkar DS Vaidya AB *In vitro *action of Dadima (*Punica granatum *Linn.) against microorganisms involved in human gastrointestinal infections - isolation and identification of tannins Journal of Research in Ayurveda and Siddha 1993 14 154 164

[B49] Mukherjee PK Saha K Murugesan T Mandal SC Pal M Saha BP Screening of anti-diarrhoeal profile of some plant extracts of a specific region of West Bengal, India J Ethnopharmacol 1998 60 85 89 10.1016/S0378-8741(97)00130-X 9533436

[B50] Oben JE Assi SE Agbor GA Musoro DF Effect of *Eremomastax speciosa *on experimental diarrhoea African J Trad Complemen Altern Med 2006 3 95 100 http://www.africanethnomedicines.net/v3n1obenetal.pdf

[B51] Rao VSN Santos FA Sobreika TT Souza MF Melo LL Silveira ER Investigations on the gastroprotective and antidiarrhoeal properties of ternatin, a tetramethoxyflavone from *Egletes viscose* Planta Med 1997 63 146 149 10.1055/s-2006-957632 9140229

[B52] Tona L Kambu K Mesia K Cimanga K Apers S De Bruyne T Pieters L Totte J Vlietinck AJ Biological screening of traditional preparations from some medicinal plants used as antidiarrhoeal in Kinshasa, Congo Phytomedicine. 1999 6 1 59 66 10.1016/S0944-7113(99)80036-1 10228613

[B53] Venkatesan N Thiyagarajan V Narayanan S Arul A Raja S Kumar SGV Rajarajan T Perianayagam JB Anti-diarrhoeal potential of *Asparagus racemosus *wild root extracts in laboratory animals J Pharm Pharm Sci 2005 8 1 39 45 http://www.ualberta.ca/~csps/JPPS8(1)/J.Perianayagam/asparagus.pdf 15946596

[B54] Rosales-Reyes T de la Garza M Arias-Castro C Rodríguez-Mendiola M Fattel-Fazenda S Arce-Popoca E Hernandez-Garcia S Villa-Trevino S Aqueous crude extract of Rhoeo discolor, a Mexican medicinal plant, decreases the formation of liver preneoplastic foci in rats J Ethnopharmacol 2008 115 381 386 10.1016/j.jep.2007.10.022 18063494

[B55] Ojewole JA Awe EO Chiwororo WD Antidiarrhoeal activity of *Psidium guajava *Linn. (Myrtaceae) leaf aqueous extract in rodents J Smooth Muscle Res 2008 44 195 207 10.1540/jsmr.44.195 19234374

[B56] Etuk EU Francis UU Acute toxicity and efficacy of *Psidium guajava *leaves water extract on *Salmonella typhi *infected Wistar rats Pak J Biol Sci 2003 6 195 7 10.3923/pjbs.2003.195.197

[B57] Grover IS Bala S Studies on antimutagenic effects of guava (*Psidium guajava*) in *Salmonella typhimurium* Mutat Res 1993 300 1 1 3 10.1016/0165-1218(93)90133-X 7683763

[B58] Teixeira Rdo Camparoto ML Mantovani MS Vicentini VEP Assessment of two medicinal plants, *Psidium guajava *L. and *Achillea millefolium *L., in *in vitro *and *in vivo *assays Genet Mol Biol 2003 26 551 555 10.1590/S1415-47572003000400021

[B59] Anonymous Quercetin Alt Med Rev 1998 3 140 143 http://www.thorne.com/altmedrev/.fulltext/3/2/140.pdf 9577251

[B60] Lozoya X Meckes M Abou-Zaid M Tortoriello J Nozzolillo C Arnason JT Quercetin glycosides in *Psidium guajava *L. leaves and determination of a spasmolytic principle Arch Med Res 1994 25 11 15 8019108

[B61] Morales MA Tortoriello J Meckes M Paz D Lozoya X Calcium-antagonist effect of quercetin and its relation with the spasmolytic properties of *Psidium guajava *L Arch Med Res 1994 25 17 21 8019109

[B62] Lutterodt GD Inhibition of gastrointestinal release of acetylcholine by quercetin as a possible mode of action of *Psidium guajava *leaf extracts in the treatment of acute diarrhoeal disease J Ethnopharmacol 1989 25 235 247 10.1016/0378-8741(89)90030-5 2747259

[B63] Galvez J Zarzuelo A Crespo ME Utrilla MP Jimenez J Spiessens C de Witte P Antidiarrhoeic activity of *Scleroarya birrea *bark extract and its active tannin constituent in rats Phytother Res 1991 5 276 278 10.1002/ptr.2650050611

[B64] Galvez J Crespo ME Jimenez J Suarez A Zarzuelo A Antidiarrhoeic activity of quercitrin in mice and rats J Pharm Pharmacol 1993 45 157 159 8095537 10.1111/j.2042-7158.1993.tb03706.x

[B65] Galvez J Zarzuelo A Crespo ME Lorente MD Ocete MA Jimenez J Antidiarrhoeic activity of *Euphorbia hirta *extract and isolation of an active flavonoid constituent Planta Med 1993 59 333 336 10.1055/s-2006-959694 8372151

[B66] Otshudi AL Vercruysse A Foriers A Contribution to the ethnobotanical, phytochemical and pharmacological studies of traditionally used medicinal plants in the treatment of dysentery and diarrhoea in Lomela area (DRC) J Ethnopharmacol 2000 71 411 423 10.1016/S0378-8741(00)00167-7 10940578

[B67] Mavar-Manga H Haddad M Pieters L Baccelli C Penge A Quetin-Leclercq J Anti-inflammatory compounds from leaves and bark of *Alchornea cordifolia *(Schumach. & Thonn.) Mull. Arg J Ethnopharmacol 2008 115 25 29 10.1016/j.jep.2007.08.043 17942256

