# Supplementary material for: Newer insights into the mechanism of action of Psidium guajava L. leaves in infectious diarrhoea
Source: BMC Complement Altern Med. 2010 Jun 28;10:33. doi: 10.1186/1472-6882-10-33 (PMC2911405; doi:10.1186/1472-6882-10-33)

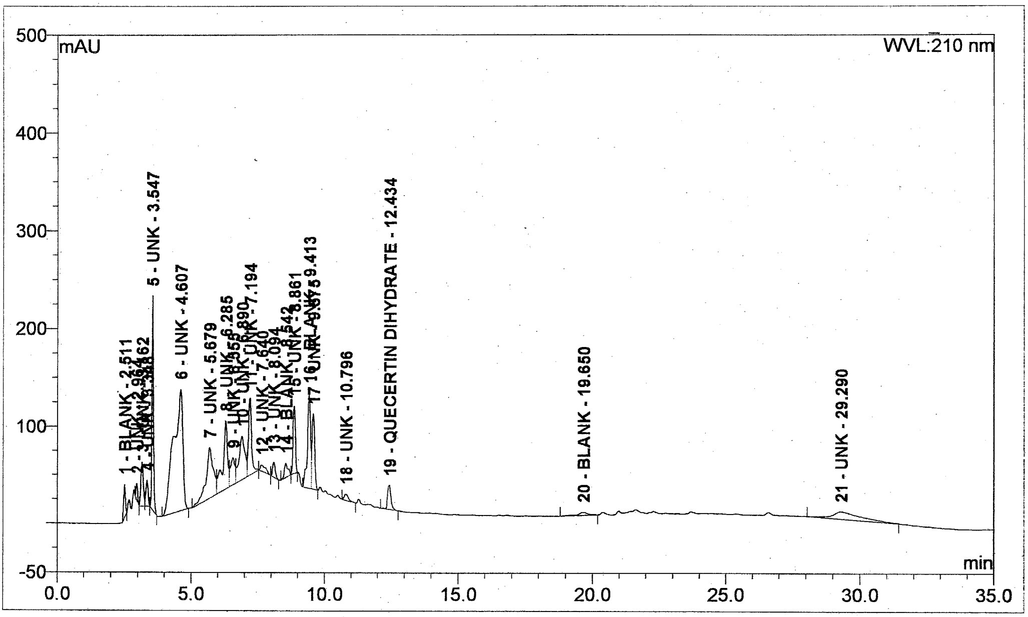

No.	Ret.Time	Peak Name	Height	Area	Rel.Area	Match
	min		mAU	mAU*min	%	
1	2.511	BLANK	33.481	2.422	1.30	997
2	2.964	UNK	16.872	1.219	0.65	987
3	3.162	UNK	45.534	4.761	2.55	993
4	3.348	UNK	26.499	2.421	1.29	995
5	3.547	UNK	219.315	15.177	8.12	998
6	4.607	UNK	125.014	50.569	27.04	999
7	5.679	UNK	52.164	16.088	8.60	966
8	6.285	UNK	69.949	14.450	7.73	989
9	6.555	UNK	27.824	5.486	2.93	997
10	6.890	UNK	44.862	11.674	6.24	996
11	7.194	UNK	79.193	10.895	5.83	977
12	7.640	UNK	7.080	1.678	0.90	911
13	8.094	UNK	16.094	1.851	0.99	965
14	8.542	BLANK	15.090	2.261	1.21	952
15	8.861	UNK	70.052	7.398	3.96	818
16	9.413	BLANK	98.469	13.546	7.24	925
17	9.575	UNK	77.667	9.384	5.02	996
18	10.796	UNK	6.309	1.124	0.60	851
19	12.434	QUECERTIN DIHYDRATE	24.698	3.732	2.00	992
20	19.650	BLANK	3.287	1.400	0.75	712
21	29.290	UNK	7.436	9.463	5.06	938
Total:			1066.890	187.000	100.00	

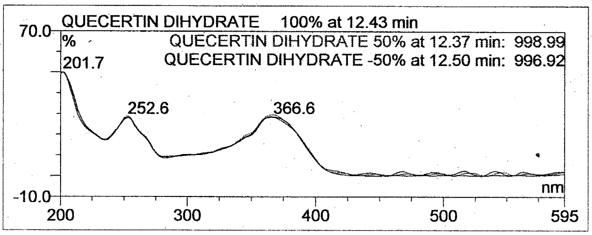

Supplement: Additional file 1 — HPLC profile of the decoction of P. guajava. HPLC profile of the acid hydrolysed decoction of P. guajava shows the presence of the standard reference compound quercetin. [file 1472-6882-10-33-S1.PDF]
